# Editorial: Twenty Years After Implicit Association Test: The Role of Implicit Social Cognition in Human Behavior

**DOI:** 10.3389/fpsyg.2022.919658

**Published:** 2022-05-26

**Authors:** Zheng Jin, Kimihiro Shiomura, Christina Bermeitinger, Xiangru Zhu, Maria Clara P. De Paula Couto, Lili Wu, Pamela Baess

**Affiliations:** ^1^Henan International Joint Laboratory of Psychological Data Science, Zhengzhou Normal University, Zhengzhou, China; ^2^International Joint laboratory of Behavior and Cognitive Science, Zhengzhou Normal University, Zhengzhou, China; ^3^Department of Communication Studies, Ferris University, Yokohama, Japan; ^4^Institute of Psychology, University of Hildesheim, Hildesheim, Germany; ^5^Institute of Cognition, Brain and Health, Henan University, Kaifeng, China; ^6^Institute of Psychology, Friedrich Schiller University Jena, Jena, Germany; ^7^CAS Key Laboratory of Mental Health, Institute of Psychology, Chinese Academy of Sciences, Beijing, China; ^8^Department of Psychology, University of Chinese Academy of Sciences, Beijing, China

**Keywords:** implicit measures, Implicit Association Test (IAT), multinomial model, attitude-behavior relationships, association

Implicit measures, marked by the Implicit Association Test (IAT) born 25 years ago, have been expanded far beyond social psychology to diverse applications (alcohol-related cognitions, Wiers et al., 2002; and drug therapy, Wiers et al., 2007; e.g., medical treatment, Green et al., 2007). However, despite the widespread popularity of implicit measures such like IAT, controversies remain about theoretical, methodological, and empirical questions, and the contribution of implicit measures to solving real-world problems. Whatever the ultimate answers to these questions, however, it seems hard to imagine a future of the social psychology field where implicit measures will not play a major role. A concerted effort to address unresolved issues may help to move the field forward, and the articles in this Special Issue may provide some helpful directions in this regard.

This Special Issue starts with a focus on novel applications of the IAT. One article provides evidence for an implicit association between the direct gaze and the concept of closeness, which inspires future research to examine social function of other body signals (Cui et al.); Another article devoted to examining the relation between ostracism and automatic aggression, where most previous research on the “ostracism-aggression” link has focused on controlled processes (Zhang et al.).

Subsequent contributions reviewed research adopting implicit methodology in the educational domain. Over the last 10 years, several studies have been conducted in different countries, involving in- and pre-service teachers and investigating their attitudes toward different student groups, the greatest caveat is, however, formed by the lack of research regarding the association between implicit attitudes, teacher expectations and behavior and, in turn, student outcomes. Pit-ten Cate and Glock's meta-analysis validates the distinction between the constructs of implicit and explicit attitudes and indicate that both should be considered in attitude research. They conclude that future research should investigate possible links between implicit attitudes and other constructs more systematically to test the suitability of the theoretical models concerning attitudes within the educational domain and to gain better understanding of underlying mechanisms that contribute to the educational inequalities that different groups of student's experience.

With implicit measures like the IAT, researchers hoped to finally be able to bridge the gap between self-reported attitudes on one hand and behavior on the other. However, after 20 years of research and several meta-analyses, we have to conclude that neither the IAT nor its derivatives have fulfilled these expectations. Their predictive value for behavioral criteria is weak and their incremental validity over and above self-report measures is negligible. Rothermund et al. present an overview of explanations for these unsatisfactory findings and delineate promising ways forward. As they indicated, one of the reasons for which the IAT and its derivatives did not succeed in closing the attitude-behavior gap is that implicit task performance is affected by a variety of non-associative processes such as stimulus recoding (e.g., Meissner and Rothermund, 2013; Jin, 2016); overcoming biased associations and the ability to detect correct responses on the task (for review, Calanchini et al., 2018). Another contribution from Sherman and Klein describes four theoretical and methodological problems revolving around assumptions made about the relationships among measures (indirect vs. direct), constructs (implicit vs. explicit attitudes), cognitive processes (e.g., associative vs. propositional), and features of processing (automatic vs. controlled). These assumptions have confused our understandings of exactly what we are measuring, the processes that produce implicit evaluations, the meaning of differences in implicit evaluations across people and contexts, the meaning of changes in implicit evaluations in response to intervention, and how implicit evaluations predict behavior. Meissner et al. and Sherman and Klein offer outlooks and conceptual recommendations for future implicit attitude research. Consistent with their suggestion that using sophisticated analysis tools to identify processes associated with implicit attitude malleability, variability, and behavior prediction, Reichardt et al. investigate the validity of the measure of stereotypic impression formation, the stereotype misperception task (SMT), together with a multinomial model that quantitatively disentangles the contributions of stereotype activation and application to responses in the SMT. The authors hope to advance research on stereotyping by providing a measurement tool that separates multiple processes underlying impression formation.

Nguyen-Phuong-Mai conducted a preliminary study of reviewing and exploring bias strategies using a framework of a different discipline: change management. Twenty years since IAT, many studies have focused on strategies to reduce this biased tendency. However, there is little evidence that a reduction in implicit biases will translate into changes in behaviors (Forscher et al., 2019). The author uses a theoretical framework of change management to explore suggestions from interdisciplinary studies that could be potential to improve and develop bias strategies that results in a change in behavior.

The Special Issue received 20 manuscripts and to a certain extent attracted attention; the acceptance rate of just one-third reflects the rigorous review criterion. Admittedly, the field is still facing a number of unresolved challenges and open questions, including the role of methodological factors in the interpretation of results obtained with implicit measures, the meaning and implications of their low temporal stability, their presumed value in predicting behavior and other psychological outcomes, and questions pertaining to the updating and change of underlying mental representations. Nevertheless, we offer some guidance and at least a few more pieces of the puzzle for the next generation of research using implicit measures.

## Author Contributions

ZJ drafted the paper. All authors provided critical comments and additions and approved it for publication.

## Funding

This study was supported by Program for Science and Technology Development of Henan Province (222102310686) and Talents Program of the Ministry of Science and Technology (G2021026014L and DL2021026005L).

## Conflict of Interest

The authors declare that the research was conducted in the absence of any commercial or financial relationships that could be construed as a potential conflict of interest.

## Publisher's Note

All claims expressed in this article are solely those of the authors and do not necessarily represent those of their affiliated organizations, or those of the publisher, the editors and the reviewers. Any product that may be evaluated in this article, or claim that may be made by its manufacturer, is not guaranteed or endorsed by the publisher.
